# Thioglycolic Acid FTIR Spectra on Ag_2_S Quantum Dots Interfaces

**DOI:** 10.3390/ma13040909

**Published:** 2020-02-18

**Authors:** Tamara Kondratenko, Oleg Ovchinnikov, Irina Grevtseva, Mikhail Smirnov, Oksana Erina, Vladimir Khokhlov, Boris Darinsky, Elena Tatianina

**Affiliations:** 1 Department of Optics and Spectroscopy, Voronezh State University, 394018 Voronezh, Russia; ovchinnikov_o_v@rambler.ru (O.O.); grevtseva_ig@inbox.ru (I.G.); smirnov_m_s@mail.ru (M.S.); 2 Federal State Budget Educational Institution of Higher Education “Voronezh State University of Engineering Technologies”, 3394036 Voronezh, Russia; 3 Department of Analytical Chemistry, Voronezh State University, 394018 Voronezh, Russia; olesja3112@yandex.ru (O.E.); khokhlov@chem.vsu.ru (V.K.); darinskii@mail.ru (B.D.); 4 Department of Physics, Voronezh State Technical University, 394006 Voronezh, Russia; elena.tatianina@yandex.ru

**Keywords:** thioglycolic acid (TGA), Ag_2_S quantum dots, FTIR spectra, luminescence, photodegradation, dimer, ionic form

## Abstract

The mechanism features of colloidal quantum dots (QDs) passivation with thioglycolic acid molecules (TGA) for cases of different luminescent properties is considered using FTIR. This problem is considered based on FTIR spectra analysis for various ionic forms of TGA. Experimental TGA molecules FTIR spectra is interpreted, basing on the data on modeling of TGA vibrational modes, realized in the framework of density functional method (DFT /B3LYP/6-31+G(d)) taking into account the vibrations anharmonicity of every functional group. This approach provides a significant improvement in the agreement between the experimental and calculated data. FTIR spectra of Ag${}_{2}$S/TGA QDs with exciton and recombination luminescence are differ from each other and B “freeB” TGA molecules. The ν(S−H) TGA peak (2559 cm${}^{-1}$) disappears in FTIR spectra of Ag${}_{2}$S/TGA QD samples. This fact indicates the interactions between TGA thiol group and dangling bonds of Ag${}_{2}$S nanocrystals. Ag${}_{2}$S QDs passivation with TGA molecules leads to emergence $\nu_{as}$(COO${}^{-}$) (1584 cm${}^{-1}$) and $\nu_{s}$(COO${}^{-}$) (1387 cm${}^{-1}$) peaks. It indicates TGA adsorption in ionic form. For Ag${}_{2}$S/TGA QDs with exciton luminescence we observed (a) significant low-frequency shift of $\nu_{s}$(COO${}^{-}$) peak from 1388 cm${}^{-1}$ to 1359 cm${}^{-1}$ and high-frequency shift of $\nu_{as}$(COO${}^{-}$) peak from 1567 cm${}^{-1}$ to 1581 cm${}^{-1}$; (b) change in the ratio of intensities of $\nu_{as}$(COO${}^{-}$) and $\nu_{s}$(COO${}^{-}$) vibrations. This feature is caused by the change in the symmetry of TGA molecules due to passivation of Ag${}_{2}$S quantum dots.For Ag${}_{2}$S/TGA QDs with recombination luminescence, the insignificant high-frequency shift of 7–10 cm${}^{-1}$ for $\nu_{as}$ (COO${}^{-}$) at 1567 cm${}^{-1}$ and low-frequency shift of 3–5 cm${}^{-1}$ for $\nu_{s}$ (COO${}^{-}$) at 1388 cm${}^{-1}$, probably caused by the interaction of thiol with Ag${}_{2}$S surface is observed. Using FTIR spectra, it was found that IR luminescence photodegradation is also accompanied by changes in the thioglycolic acid molecules, which capped Ag${}_{2}$S QDs. In the case of Ag${}_{2}$S QDs with exciton luminescence, the degradation process is non-reversible. It is accompanied by TGA photodegradation with the formation of α-thiol-substituted acyl radical (S-CH${}_{2}$-CO${}^{•}$) TGA.

## 1. Introduction

The problem of obtaining functional nanomaterials based on semiconductor colloidal quantum dots (QDs) with specific luminescent properties is relevant, primarily for creating luminescent sensors for biology, medicine, chemistry, geology, etc. [1,2,3,4,5,6,7,8,9]. The coating of nanocrystal interfaces with size of several nanometers with various ligands prevents their agglomeration. The molecule functional groups are selected with aim of the removing or minimizing the concentration of QDs surface dangling bonds due to interaction with them.This provide optimal luminescent properties of colloidal quantum dots.

Thioglycolic acid (TGA) is actively and successfully used as passivator in the synthesis of colloidal quantum dots and core/shell systems [4,5,6,7,8,9,10,11,12,13,14,15,16,17]. Many important problems of nanophotonics are closely related to the interaction mechanism of QDs with organic matrix, which ensure the nanostructures formation with special composition and stoichiometry. In particular, the choice of passivator molecules determines the QDs luminescent properties [18,19]. For optimization of the interface passivation conditions it is necessary to know interaction mechanism of TGA molecules and QDs interfaces. TGA bifunctionality is a specific feature of these molecules, acting as QDs passivators [4,20,21,22]. These molecules can interact with dangling bonds QD through both the thiol and carbonyl groups [6,7,8,13,21,22,23]. It should be taking into account that TGA in a solution is capable to self-association, as well as the formation of other polynuclear complex forms (sodium salt, anion and dianion, etc.) [7,15,24,25,26].

To date, the most frequent studies of QDs interaction mechanisms with organic molecules are based on UV-Vis absorption and luminescence spectra [27,28,29].These data are used for interaction type proposing. It can be covalent binding or electrostatic absorption of stabilizer molecules. In the meantime, FTIR spectroscopy data have been found to significantly refine and specify the intermolecular interactions mechanisms, allowing us to find stabilizer groups that are active in the interaction and propose an intermolecular interactions model in the nanocrystal-stabilizer system [4,12,13,14,15,16,17,30]. Such models are not universal, they depend on QDs material and used stabilize. Therefore, they require refinement in each specific case [9,13].

The most informative method to investigation the interaction type and passivator molecules structure is Fourier-transform infrared (FTIR) spectroscopy. Using this method requires unambiguous understanding TGA FTIR spectra in the initial solution, using for passivating QDs. The interpretation of TGA molecules FTIR spectra is ambiguous [3,5,6,10,12,14,21,22,23,24,25,26,30,31,32,33,34,35,36,37,37]. This provides to ambiguity in FTIR spectra interpretation of TGA molecules and passivation mechanisms of semiconductor colloidal quantum dots, as well as plasmon nanoparticles.

This work is devoted to the analysis of passivation mechanisms of Ag${}_{2}$S quantum dots with TGA molecules with various luminescent properties. The solution to this problem is based on FTIR spectra interpretation for various ionic forms of TGA molecules, based on the results of modeling the FTIR vibration modes of various ionic forms and TGA dimers, obtained in the framework of density functional method (DFT/B3LYP/6-31+G(d)) using the Gaussian-03 software package.

## 2. Methods of Investigation

The studied samples were colloidal solutions of Ag${}_{2}$S QDs, passivated with TGA and aqueous TGA solutions with different pH. Colloidal Ag${}_{2}$S/TGA QDs were prepared by the aqueous synthesis technique [18,19]. All reagents (Na${}_{2}$S, AgNO${}_{3}$, TGA, NaOH) purchased from Sigma-Aldrich were of high purity. In the first approach, Na${}_{2}$S aqueous solution was used as the sulfur source. TGA molecules, in this case, were mainly used for the interface passivation. In the synthesis, we used an Ag${}^{+}$/TGA precursor solution (200 mL) obtained by mixing AgNO${}_{3}$ (2.6 mmol) and TGA (2.6 mmol) with subsequent raising of pH to 10 by addition of a 1M NaOH solution. After that, with constant stirring, 50 mL of a Na${}_{2}$S aqueous solution were added using a peristaltic pump. The solution in the reactor changed color from light yellow to dark brown. Thus, Ag${}_{2}$S QDs with a concentration of 2 × 10${}^{-5}$ mol QDs/L were formed in water. The synthesis was carried out at a temperature of 30 ${}^{\circ }$C. [AgNO${}_{3}$]:[TGA]:[Na${}_{2}$S] molar ratio, in this case, was 1:1.1:0.33.

In the second approach to the synthesis of Ag${}_{2}$S QDs, a TGA aqueous solution was used as the only sulfur source. TGA molecules also acted as the passivating agent. In this synthesis, Ag${}_{2}$S QD samples were obtained in the same way as described above, but without using Na${}_{2}$S. [AgNO${}_{3}$]:[TGA]:[Na${}_{2}$S] molar ratio, in this case, was 1:0.9:0.

These approaches to the synthesis make it possible to obtain ensembles of colloidal quantum dots that are fundamentally different in their physical properties. The structural and spectral properties of such samples are considered in detail in works [18,19]. Ag${}_{2}$S QDs in solution was $2\times 10^{-5}$ mol QDs/L. With the ratio of ion concentrations [AgNO${}_{3}$]:[TGA]:[Na${}_{2}$S] ranging from 1:0.9:0 to 1:1.1:0.33, Ag${}_{2}$S particles are formed with sizes 2.0 nm and 2.5 nm, respectively. In this work, we consider the relation of luminescent properties with passivation mechanism of QDs interface with TGA molecules.

**Figure 1:**
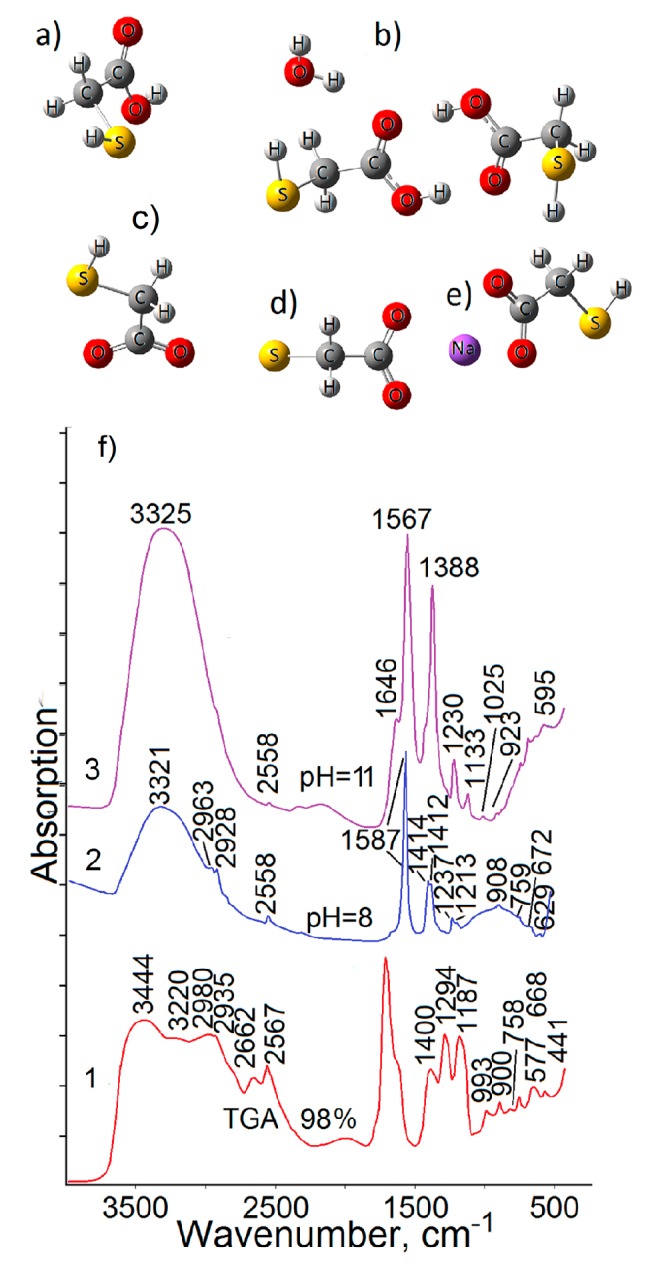
Structure of TGA, its associates and ionic forms and their FTIR spectra. (a) monomer; (b) cyclic dimer and water molecule; (c) dTGA; (d) ddTGA; (e) sodium thioglycolate; (f) FTIR spectra of TGA solutions, recorded at different pH values.

Solutions with various pH values were obtained by dropwise introducing 1 M NaOH solution into pure TGA solution reaching the required value. pH value was controlled by a pH-150M pH meter (Russia). 0.15 mL of obtained solution was applied on the windows using measuring pipette. pH values were picked out with aim to obtain various TGA ionic forms (deprotonated (dTGA), double deprotonated (ddTGA)). Their structures are presented in Figure 1. We used known values pK1 = 3.48, pK2 = 10.11 [38]. At 8 acid molecules present in solution in ionic form (dTGA). For pH > 11 there is double deprotonated form (ddTGA).

Analysis of various TGA ionic forms was carried out using FTIR spectra. They were recorded on Tensor 37 FTIR spectrometers with a beam splitter from KBr, DTLS detector (Bruker Optik GmbH) under strictly constant conditions in the region of 400–4000 cm${}^{-1}$. The spectrometer control, recording and adaptation of spectra were performed using OPUS 7.0 software.

0.15 mL of obtained solution was applied to the cell window using measuring pipette. The solution itself is one of the most significant peaks in the infrared region. The window surface was uniformly wetted and dried in air stream, heated to 40–50 ${}^{\circ }$C. TGA with high purity, obtained from Sigma-Aldrich company were used in this investigation. In preliminary studies dried TGA solution was prepared on windows of KCl and CaF${}_{2}$. The authors took into account that in the conduction of the samples preparation with this method TGA sodium salt is formed.

Computer modeling of FTIR vibrational modes was performed for various TGA molecule configurations (monomer (Figure 1a); cyclic dimer and water molecule (Figure 1b); dTGA (Figure 1c); ddTGA (Figure 1d); sodium thioglycolate (Figure 1e)). Estimations were done by the density functional method, DFT/B3LYP/6- 31+G(d) using GAUSSIAN-09 software package [39]. In the case of various ionic forms complexes of TGA and water molecules the molecular geometry was regulated in accordance with the minimum potential energy of all stereoisomers, calculated previously.

The approach to calculate TGA FTIR spectra within this basis framework supposes the use of a harmonic approximation. The obtained results should be predictive. Therefore, the method was developed for taking into account the effect of anharmonicity on the vibrational frequencies value, using the technique, based on the calculation of the system energy characteristics as a function of atoms coordinates, allowing to present the field of forces acting between the atoms [40].

Calculating the energy of this configuration in the framework of GAUSSIAN-09 software package allows us to find the dependence of the system potential energy on the parameter q for each mode. Further, this dependence is approximated by the equation

(1) $$V\left(q\right)=V\left(0\right)+\frac{c}{2}q^{2}-\alpha {\left(\frac{q}{a}\right)}^{3}$$

where V(q) is the potential energy of atomic displacements of the chosen mode, presented as the generalized coordinate function *q* of this mode, *c* is the effective stiffness of the chosen mode, α is the third-order anhormonicity coefficient, *a* is the characteristic displacements value, determined by equation

(2) $$\alpha ={\left(\frac{\hbar \omega }{c}\right)}^{\frac{1}{2}}$$

The effective stiffness (*c*) and anharmonicity coefficient (α) are determined using data on energy of V(0), V(q) and V(−q), found for appropriately selected displacements *q*

(3) $$c=\frac{V(q)+V(-q)-2V(0)}{q^{2}}$$

(4) $$\alpha =\frac{a^{3}}{2q^{3}}\left(V\left(-q\right)-V\left(q\right)\right)$$

Similar equations were used in [41] for the entire set of vibrational modes. Detailed estimates of accuracy in this method were given in [42]. In the present work, the criterion for choosing the q value was the equation

(5) $$\frac{\left(V(q)+V(-q)\right)}{2}-V\left(0\right)=\hbar \omega$$

It corresponds to the vibration amplitudes with the energy of the quantum oscillator ground state. Using the well-known results of a quantum-mechanical calculation of the anharmonic oscillator energy levels [43] we find the relative frequency change of vibrations

(6) $$\frac{\Delta \omega }{\omega }=-\frac{15}{2}{\left(\frac{\alpha }{\hbar \omega }\right)}^{2}$$

Using equations (1), (3)–(5) we determine

(7) $$\frac{\Delta \omega }{\omega }=-\frac{15}{16}\left(\hbar \omega \right)\frac{\Delta V^{2}}{V^{3}}$$

where $\Delta V=\frac{1}{2}\left(V\left(-q\right)-V\left(q\right)\right)$, $V=\frac{1}{2}\left(V\left(-q\right)-V\left(q\right)-2V\left(0\right)\right)$

Equation (7) shows that the relative change in the frequency, associated with third-degree anharmonicity is negative and proportional to the frequency. The proportionality coefficient changes during the transition from one vibrational mode to another. In particular, the contribution from nonlinearity is relatively large for localized modes, in which the bond lengths between neighboring atoms vary noticeably, compared to the deformation modes, for which the distances between neighboring atoms very little. Although that only one nonlinearity coefficient is taken into account in (6), this approach leads to a significant improvement in the agreement between the experimental and calculated modes in the region of stretching vibrations of CO and OH bonds.

Optical absorption spectra were obtained by a USB2000+ spectrometer (OceanOptics, USA) with a USB-DT radiation source (Ocean optics).

Investigations of luminescence spectra of colloidal QDs were realized, using an automated spectral complex, based on a diffractive monochromator MDR-23 (LOMO). A highly stable low-noise photodiode PDF10C/M (ThorlabsInc., USA) with a built-in amplifier was used as photodetector in the near-IR region. For luminescence excitation, we used an NDV7375 laser diode (Nichia, Japan) with 405 nm emission and PM-G80 (CST, China) laser module with 532 nm emission and 100 mW optical power. Presented results were obtained at room temperature.

## 3. Results and Discussion

### 3.1. FTIR Spectra of TGA Water Solutions

First of all, FTIR spectra of various ionic forms of TGA were analyzed. Figure 1f shows FTIR spectra of TGA aqueous solutions under conditions of different pH values. Table 1 summarizes the data on TGA vibrational modes, obtained from FTIR spectra and calculation results. They are necessary for the characteristic modes analysis.

The spectra of all studied samples include the noticeable bands set, whose position, intensity and full width at half maximum experience significant changes during increasing in pH value.

The largest changes were obtained in the region of both bound stretching modes (3000–3500 cm${}^{-1}$) and stretching and bending vibrations of carboxyl functional groups (1700–1100 cm${}^{-1}$).

FTIR spectrum of 98% TGA solution (pH = 2) has a complex band at 2500–3600 cm${}^{-1}$ (Figure 1f, curve 1, Table 1). In the high-frequency spectrum region, a broad band with peak at 3445 cm${}^{-1}$ and feature in the region of 3220 cm${}^{-1}$ was found. These bands belong to –OH groups vibration, involved in H-bonds. At the same time, the peak at 3220 cm${}^{-1}$ belongs to –OH groups vibration in COOH fragment, participating in the formation of TGA dimers. Another broad band with peak at 3445 cm${}^{-1}$ can be attributed to –OH groups of water molecules, located near TGA molecules and promoting the formation of H-bond bridges, including dimerization process, which are less bonded with TGA molecules [7,12,25,26,34]. This hypothesis is supported by the disappearance of the first band (3220 cm${}^{-1}$) with increase in pH value, as well as a decrease in frequency value from 3445 cm${}^{-1}$ to 3325 cm${}^{-1}$ with decrease in TGA dimers fraction. In addition to these two peaks there is a band with peak at 2980 cm${}^{-1}$ in FTIR spectra of concentrated TGA solutions. This peak also belongs to the stretching vibrations of OH-bonded groups in TGA molecule. Apparently, TGA dimers formation affects these vibrations symmetry and their group character. This hypothesis is confirmed by the calculation results. For the case of TGA dimers, the appearance of two modes belonging to the stretching vibrations of OH-bonded groups (3086 cm${}^{-1}$ and 2987 cm${}^{-1}$, see Table 1) is observed. The addition of water molecules near TGA leads to the appearance of a peak at 3490–3500 cm${}^{-1}$ (Table 1).

It should be noted that the band at 3440–3450 cm${}^{-1}$ shifts to the low-frequency region (3315–3325 cm${}^{-1}$) with increasing in pH value, significantly changing TGA molecules structure, breaking cyclic dimers formation, forming TGA sodium and anion molecules forms. This fact also confirms our conclusion that the peak at 3445 cm${}^{-1}$ belongs to –OH groups vibrations in water molecules, located near TGA molecules and promoting the H-bond bridges formation, including dimerization process. TGA molecules deprotonation and dimer destruction follow to changing the interaction character with –OH groups in water molecules. According to spectrum, H-bond strength increases. It is manifested in the hypsochromic shift of the band from 3440–3450 cm${}^{-1}$ to 3315–3325 cm${}^{-1}$.

The band with peak at 2935 cm${}^{-1}$ and feature near 2882 cm${}^{-1}$, appeared as single low-intensity band in the condition of higher pH value is attributed to the asymmetric and symmetric stretching vibrations of CH${}_{2}$ groups, respectively [15,24,25,26,34]. When pH value increases, the changes in the position of these vibration modes do not exceed 6–10 cm${}^{-1}$. This fact indirectly confirms the structural TGA molecule transformations and effect of –OH solvent groups.

The next most intense peak at 2567 cm${}^{-1}$ is related to the SH stretch of TGA molecules. It is in agreement with calculation results. This peak position some shifts with increasing in pH value. According to the calculation data, this band position is also sensitive to TGA dimers formation (shift from 2576 cm${}^{-1}$ to 2559 cm${}^{-1}$) (Table 1). The dimers destruction and TGA molecule deprotonation, as well as an increase in the concentration of OH-ions in solution explain the behavior of this band. This hypothesis is supported by a significant decrease in this band intensity with increasing in pH value that corresponds to increasing in fraction of double-deprotonated molecules.

The peak at 2662 cm${}^{-1}$ is another distinctive band in this FTIR spectrum region. This peak is observed for 98% TGA solution. Apparently, the presence of TGA dimers is also confirmed by the presence of this distinctive band at 2662 cm${}^{-1}$, which is characteristic of carboxylic acids dimmers [25,26,31,32,33]. However, this band is not due to the SH stretch with another symmetry in a dimer, which are differ from TGA monomer, since the increase in pH value this peak. It is important that this band does not exist in the calculated spectra, for which the anharmonic effect was not laid down at the base determination stage. The complex anharmonic nature of this peak was confirmed in [25]. In this work, this band is related to a complex vibration, caused by overtones and combinations of 1294 and 1400 cm${}^{-1}$ bands due to the interacting C-O stretching and in-plane COH bending vibrations. The attribution of this band to the SH stretches is also questionably. It disappears with increase in pH solution value. And in these conditions, the SH bending vibrations in the low-frequency region (950–1030 cm${}^{-1}$) (Figure 1f). Note that the calculations in the framework of models considering Fermi coupling between the OH stretch and nearly resonant combination bands for carboxylic acid dimer show the band at 2600–2700 cm${}^{-1}$, characteristic of hydrogen bonds in cyclic dimer, founding its strong Fermi resonance interactions, involving the OH and CO vibrations [34].

A more intricate picture is observed in the low-frequency spectrum region. A narrow intense band with peak at 1714 cm${}^{-1}$ and feature at 1700 cm${}^{-1}$, as well as a feature near 1640 cm${}^{-1}$ are observed at 1750–1650 cm${}^{-1}$. The peak at 1714 cm${}^{-1}$ is related to the asymmetric C=O stretch. The feature at 1700 cm${}^{-1}$ is corresponded to the symmetric C=O stretch. This band structure confirms the presence of TGA molecules with dimer form in 98% solution. This band disappear with increasing in pH value. The complex band structure is explained by the breach in interaction of carbonyl groups vibrations in structures with hydrogen bond [44]. The presence of this doublet in the region of the C=O stretch was reported in [25]. It was attributed to characteristic of cyclic dimers (Figure 1b). A cyclic dimer is a structure, including two acid molecules that interact with each other due to hydrogen bonds. Atoms that form the cycle lie almost in the same plane. And the hydrogen bonds are almost linear. The calculation indicates the complex nature of discussed spectrum, which includes, in addition to the C=O stretch, the COH bending vibrations.

The low-frequency feature of discussed band at 1640 cm${}^{-1}$ is due to the bending vibrations of –OH bounded groups of H${}_{2}$O molecules in TGA solution. In the case of increasing in pH value, this band appears as a high-frequency feature near peak in the region of 1565–1587 cm${}^{-1}$, belonging to the asymmetric stretching vibrations of carboxylate anion $\nu_{as}\left(COO^{-}\right)$. It should be noted that the band parameters of bending vibrations of –OH bounded groups and its presence significantly affects the band position of asymmetric stretching vibrations of carboxylate anion $\nu_{as}\left(COO^{-}\right)$. The calculated value of the bending vibrations of –OH bounded groups of H${}_{2}$O molecules in TGA solution at 1550–1620 cm${}^{-1}$ essentially depends on the environment (TGA molecules, NaOH buffer solution). The observed inequality with experiment is also caused by the significant anharmonicity of –OH group vibrations, whose influence to the calculation is not fully taken into account.

When pH value increases to 8, both with decrease in intensity of symmetric stretch bands, TGA dimers characteristic (1700 cm${}^{-1}$) the new intense band appear at 1587 cm${}^{-1}$ and shoulder at 1380 cm${}^{-1}$, corresponding to asymmetric and symmetric stretching vibrations of carboxylate anion (COO${}^{-}$). FTIR spectrum calculations of deprotonated TGA molecule in the presence of buffer solution (NaOH) show modes with value, closed to these bands. They are 1580 cm${}^{-1}$ and 1376 cm${}^{-1}$. A subsequent increase in pH value leads to leveled of the dimer band at 1700 cm${}^{-1}$ and increase in intensity of stretch bands of carboxylate anion (Figure 1f).

In the region of 1380–1170 cm${}^{-1}$ the bands with peak at 1400 cm${}^{-1}$ and 1294 cm${}^{-1}$ disappear. They correspond to complex compound δ(COH). At the same time, lower frequency bands at 1230–1240 cm${}^{-1}$, related to the CH${}_{2}$ wagging vibrations, which are cleaner from overlapping appear. A similar picture is observed in the region of the CH${}_{2}$ twisting vibrations, overlapping with the C-O stretching vibrations at 1186 cm${}^{-1}$. An increase in solution pH value leads to the appearance of the “pure” CH${}_{2}$ twisting vibration at 1127–1169 cm${}^{-1}$.

In the region below 1100 cm${}^{-1}$, several complex composite vibrations are observed. In the mainly there are bending vibrations of CH${}_{2}$ and OCO${}^{-}$ groups in this region [15,24,25,26,34]. The band at 900 cm${}^{-1}$, observed for a 98% TGA solution corresponds to the presence of large dimers fraction. At the same time, when pH value of solution increases, this peak cannot be interpreted unambiguously due to sameness of calculated γ(OH) and $\rho (CH_{2})$ modes, characteristic of dimers and anionic acid forms. In the region of 959–1025 cm${}^{-1}$, the SH bending vibrations appear in the experimental FTIR spectra.The corresponding modes in the calculation spectra are 932–974 cm${}^{-1}$. It should be noted that when pH value increases, the intensity of discussed bands decreases significantly. This fact indicates the molecule deprotonation according to the second stage and TGA dianion formation (Figure 1). In the calculation spectra for a double-deprotonated molecule this band is absent, as well as other bands, corresponding to the vibrations of thiol group, which were observed in the cases of dimer (838, 808, 783, 746 cm${}^{-1}$). In the experimental FTIR spectrum, the SH bands appear at pH 3–8. They are absent when it increases to 10. The observed regularities in the region of bending vibrations correlates with the data, obtained for stretching vibrations and confirms the formation of the certain TGA dianions faction with increasing in solution pH value to 8. Band series include the bending vibrations of OCO${}^{-}$ groups (668, 577 cm${}^{-1}$). The band at 668 cm${}^{-1}$ corresponds to the stretching vibration. SH vibrations are observed at 758 cm${}^{-1}$.

Thus, the analysis of TGA molecules FTIR spectra, taking into account calculation results shows the following important regularities:

- for 98% TGA solution TGA FTIR spectra show bands, corresponded to dimer with maximum fraction. Dimers are formed due to H-bonds. It provides the presence in FTIR spectrum of the OH bonded stretch, including in COH acid fragment (3220 and 2980 cm${}^{-1}$) and water (3445 cm${}^{-1}$), band at 2662 cm${}^{-1}$, which characterize cyclic dimer, in-plane COH (1400 cm${}^{-1}$), the CO bending vibrations (1714 cm${}^{-1}$) and feature at 1700 cm${}^{-1}$, which also characterizes the cyclic dimer, the COH bending (1400) and C-O stretching vibrations (1187 cm${}^{-1}$ and 1294 cm${}^{-1}$), conjugated with the CH${}_{2}$ bending vibrations, out-of-plane OH bending vibration (900 cm${}^{-1}$);

- An increase in pH value leads to a significant transformation of FTIR spectrum (Figure 1f). In the high-frequency region there is a shift of peaks, corresponded to the OH stretch in water to low-frequency region from 3445 cm${}^{-1}$ to 3325 cm${}^{-1}$ with increase in pH value. It is weaker observed for the OH stretch, including in COH TGA fragment (2980 cm${}^{-1}$ to 2963 cm${}^{-1}$). At the same time, the band at 3220 cm${}^{-1}$, also related to the vibrations of OH groups, including in COH TGA fragment decreases in intensity and disappears at pH = 8. Also bands, corresponded to $\delta \left(CH_{2}\right)+\delta \left(COH\right)+\nu \left(C-O\right)$, $\nu \left(C-O\right)+tw\left(CH_{2}\right)$, $\nu \left(C-O\right)+\omega \left(CH_{2}\right)$ vibrations with peak at 1400 cm${}^{-1}$, 1187 cm${}^{-1}$, and 1294 cm${}^{-1}$, characteristic only for dimers and their specific symmetry disappear. According to calculation these modes are characteristic only for dimers and their corresponding symmetry. In addition, there is a decrease in the intensity of composed overtone band (2662 cm${}^{-1}$) with an increase in pH value at 2600–2700 cm${}^{-1}$. At the same tine, new intense peaks that relate to the asymmetric and symmetric stretching vibrations of COO${}^{-}$ in carboxylate anion (1587 and 1414 cm${}^{-1}$) appear. This behavior of FTIR spectra is caused by TGA molecule deprotonation with increasing pH value;

- In the band of the SH stretch (2568–2558 cm${}^{-1}$) we observes gradual decrease in intensity with increase in pH value to 5. Only a low-intensity band of SH stretch is observed at pH = 8 (Figure 1f). Also in the spectrum, a decrease in intensity of bands, corresponding to the SH bending vibrations (959–1025, 758, 629 cm${}^{-1}$) is observed up to their complete disappearance. This behavior is due to the increase in the fraction of double-deprotonated TGA molecules.

### 3.2. FTIR Spectra TGA Molecules, Passivating Ag${}_{2}$S QDs

**Figure 2:**
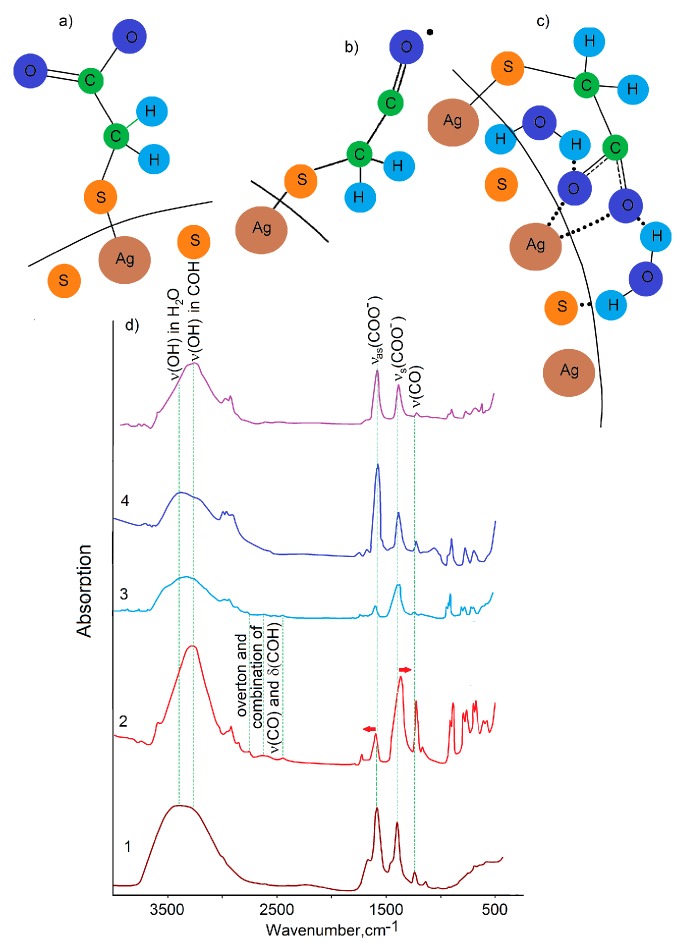
Scheme of interactions processes between TGA molecules and Ag${}_{2}$S QD surface (a, b and c). FTIR spectra of colloidal Ag${}_{2}$S/TGA QDs (d): 1 - TGA pH = 11; 2 - [AgNO${}_{3}$]:[TGA]:[Na${}_{2}$S] = 1:0.9:0; 4 - [AgNO${}_{3}$]:[TGA]:[Na${}_{2}$S] = 1:1.1:0.33 before exposure (2, 4) and after exposure (3, 5).

Using the obtained data on FTIR spectra of various ionic forms, FTIR spectra of TGA molecules adsorbed on QDs surface were interpreted. For Ag${}_{2}$S/TGA QDs synthesized at pH = 10 under different conditions and at different [AgNO${}_{3}$]:[TGA]:[Na${}_{2}$S] ratios of ion concentrations ranging from 1:0.9:0 to 1:1.1:0.33, FTIR spectra differ from each other and from the spectrum of the reference sample (pure TGA at pH = 10). The values of the wave numbers corresponding to the peaks of the characteristic frequencies in the FTIR spectra of TGA molecules are given in Figure 1f and Figure 2d.

For all FTIR spectra of the $Ag_{2}$/TGA QD samples, we observe that the peak corresponding to the stretching vibrations of the S–H group of TGA (2559 cm${}^{-1}$) vanishes, which indicates the appearance of interactions between the thiol terminal group of TGA and dangling bonds at the ${}_{2}$ QD interfaces [13,15,45,46,47,48,49].

Passivation of Ag${}_{2}$S QDs by TGA molecules gives rise to peaks of asymmetric and symmetric stretching vibrations of the carboxylic group (COO${}^{-}$) (1584 cm${}^{-1}$ and 1387 cm${}^{-1}$, respectively), which indicates adsorption of TGA molecules on the Ag${}_{2}$S interfaces in the ionic form with a free carboxylic terminal group COO${}^{-}$[13,15,45,46,47,48,49]. With increasing the concentration of [S${}^{2-}$] ions from Na${}_{2}$S during formation of Ag${}_{2}$S/TGA QDs (Figure 2d), a slight high-frequency shift of 7–10 cm${}^{-1}$ was observed for the peak of asymmetric stretching vibrations $\nu^{as}$(COO${}^{-}$) = 1567 cm${}^{-1}$ together with a low-frequency shift by 3–5 cm${}^{-1}$ for symmetric stretching vibrations $\nu^{s}$(COO${}^{-}$) = 1388 cm${}^{-1}$, probably caused by the interaction of thiol with the Ag${}_{2}$S QD surface. The intensity ratio for the peaks of asymmetric and symmetric stretching vibrations of the carboxylic group COO${}^{-}$ in the two cases under consideration (Figure 2d) corresponds to the situation when the carboxylic end groups are not attached to the surface of the Ag${}_{2}$S QD interface [13,15,45,46,47,48,49]. For the sample (Figure 2d) synthesized at the precursor ratio [AgNO${}_{3}$]:[TGA]:[Na${}_{2}$S] starting from 1:0.9:0, a significant low-frequency shift of the peak of symmetric stretching vibrations $\nu^{s}$(COO${}^{-}$) from 1388 cm${}^{-1}$ to 1359 cm${}^{-1}$ was observed together with a high-frequency shift of the peak of asymmetric stretching vibrations $\nu^{as}$(COO${}^{-}$) from 1567 cm${}^{-1}$ to 1579 cm${}^{-1}$. Such spectral shifts indicate that the COO${}^{-}$ functional groups participate in the intermolecular interactions with, e.g., Ag${}_{2}$S QD interfaces. In addition, a change in the intensity ratio for asymmetric and symmetric stretching vibrations COO${}^{-}$ was noted (Figure 2d). This feature is due to the change in the symmetry of vibrations of TGA molecules upon their adsorption by carboxylic groups on a solid substrate and is characteristic of the formation of carboxylate complexes with dangling bonds of Ag${}_{2}$S QDs [49].

Adsorption of TGA molecules on Ag${}_{2}$S QD interfaces is accompanied by the appearance of peaks at 1788 cm${}^{-1}$ and 1724 cm${}^{-1}$ (Figure 2d) with their intensity being lower than that for $\nu^{as(s)}$(COO${}^{-}$). For the samples having the maximum concentration of [S${}^{2-}$] at crystallization, these peaks disappear, and only the peak at 1743 cm${}^{-1}$ remains. These peaks are associated with vibrations $\nu^{as}$(C=O) of the COOH–group [46,49]. The occurrence of such vibrations is also a sign of the interaction of TGA molecules with Ag${}_{2}$S QDs, in which deprotonation of the thiol group is accompanied by protonation of the carboxylic group and appearance of H-bond with the Ag${}_{2}$S QD interface. Proton transfer of this kind is characteristic of TGA molecules, mainly in the acidic environment [49]. In our case, the decisive role is likely to belong to the charge of the QD interface. In addition to the mentioned peaks, weak peaks at 2766 cm${}^{-1}$, 2620 cm${}^{-1}$ were observed, which are due to the compound vibration arising from the interaction of the stretching vibrations C-O (1294 cm${}^{-1}$) and in-plane δ(C-O-H) (1400 cm${}^{-1}$). The occurrence of these peaks is uncharacteristic of TGA at pH = 10 and seems to be caused by the specifity of adsorption on Ag${}_{2}$S QDs. The complex nature of this peak was confirmed in [25,32].

For symmetric and asymmetric CH${}_{2}$ stretching vibrations in the region of 2980–2850 cm${}^{-1}$), as well as for all types of CH${}_{2}$ bending vibrations (1220–1230 cm${}^{-1}$, 1125–1133 cm${}^{-1}$, 910–750 cm${}^{-1}$[13,15,45,46,47,48,49]), variations within 5–15 cm${}^{-1}$ were detected. This feature is also a sign of the interaction between TGA and the Ag${}_{2}$S QD surface. However, CH-groups are not directly involved in the adsorption.

In each of the analyzed FTIR spectra of Ag${}_{2}$S/TGA QD samples, changes were noted for the stretching and bending vibrations of OH-bonded groups. It was found that with an increase in [S${}^{2-}$] ions concentration when using Na${}_{2}$S, a high-frequency feature arises in the range of 3350–3500 cm${}^{-1}$ (Figure 2d). This feature is related to OH-groups of water molecules located near TGA molecules and Ag${}_{2}$S/TGA QDs [15,25,32,45,46]. These OH-groups provide the H-bond bridges formation, in the case of dimerization and especially Ag${}_{2}$S QD interfaces passivation with TGA molecules. These molecules are less bonding with TGA molecules [15]. This hypothesis is also confirmed by a high-frequency shift for 30–45 cm${}^{-1}$ of the OH bending vibrations peak (1646 cm${}^{-1}$) under Ag${}_{2}$S QD interfaces passivation with TGA molecules. Similar shift indicates the occurrence of H-bonds between Ag${}_{2}$S QD interface, COOH functional groups, and H${}_{2}$O molecules.

Thus, the data obtained from FTIR spectra indicate two predominantly realized forms of TGA molecules adsorption on the Ag${}_{2}$S QD interfaces (Figure 2a,c). In the case of Ag${}_{2}$S/TGA QDs synthesis in the absent of Na${}_{2}$S, predominantly adsorption by two functional groups (thiol and carboxylic) was noted (Figure 2c). When Na${}_{2}$S is used as an additional sulfur source during Ag${}_{2}$S QDs crystallization, TGA adsorption is predominantly carried out by the thiol group (Figure 2a). It is accompanied by molecule deprotonation.

**Figure 3:**
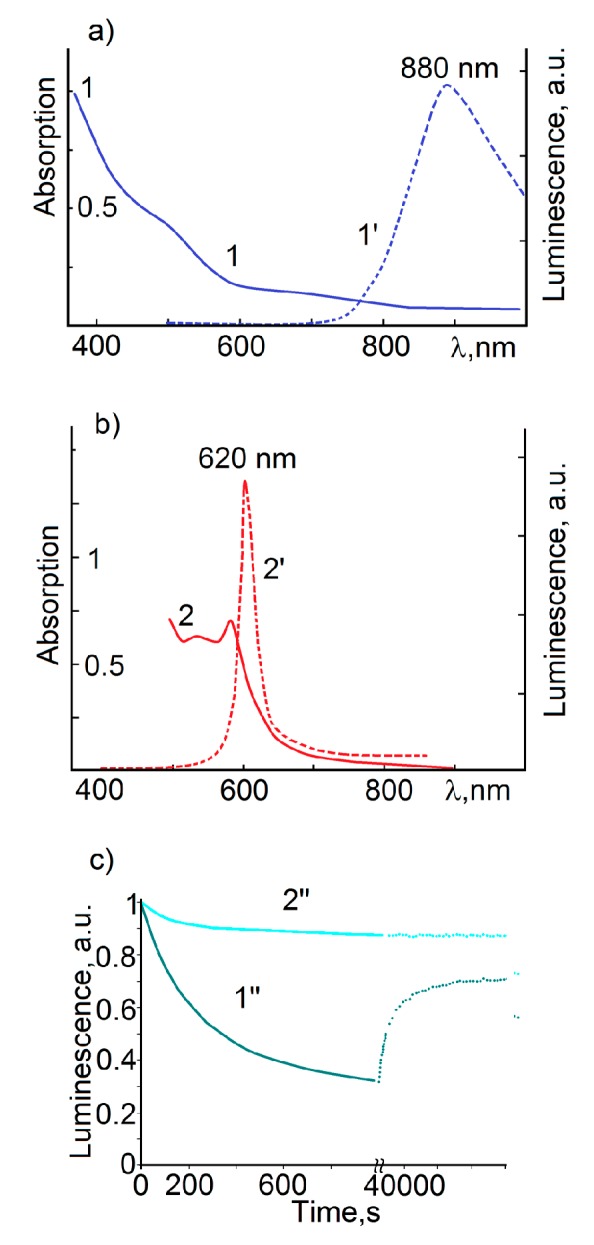
UV-Vis absorption (1, 2), photoluminescence spectra (1’, 2’) and luminescence photodegradation (1”, 2”) of colloidal Ag${}_{2}$S/TGA QDs, prepared in various synthesis condition.

Depending on the interaction mechanism of TGA molecules with QD interface, the latter have different luminescent properties. The luminescence spectrum of QDs Ag${}_{2}$S/TGA synthesized without Na${}_{2}$S, which are characterized by the adsorption of TGA molecules by two functional groups shows a narrow peak at 620 nm with a half width of 50 nm under excitation with a wavelength of 532 nm (Figure 3b). A slight Stokes shift (0.1 eV) and small half-width of the band indicate exciton luminescence for this sample. In this case, there are no recombination luminescence bands for this sample.

For colloidal Ag${}_{2}$S/TGA QDs solutions obtained using Na${}_{2}$S, when the adsorption of TGA molecules is carried out mainly by the thiol group, wider luminescence band with peak at 880 nm was observed (Figure 3a). It are distinguished by a significant Stokes shift of luminescence peak relative to the position of ground state exciton absorption. This feature indicates the recombination nature of the observed luminescence.

An important found property of Ag${}_{2}$S/TGA QDs luminescence is a decrease in its intensity with increasing in exposure time by exiting radiation. Long exposure of the samples leads to a decrease in the luminescence intensity over the entire spectrum (Figure 3c). The characteristic decay times of Ag${}_{2}$S QDs IR luminescence intensity under the exposure with a wavelength, corresponded to the exciton absorption region at an incident power of about 100 mW, are 200–300 s. Ag${}_{2}$S/TGA QDs with exciton luminescence with peak near 620 nm turned out to be the least susceptible to luminescence photodegradation. For other QDs with recombination IR luminescence in the range of 880–1000 nm, the decrease in luminescence intensity exceeded 50% under the same exposure conditions.

The photostimulated decrease in the luminescence intensity of Ag${}_{2}$S QDs turned out to be reversible mainly for samples with IR luminescence in the range of 880–1000 nm (Figure 3c, dotted line). The luminescence intensity is restored almost to its initial value in the dark within 22–24 h. The activation energy of the luminescence intensity restoring process was about 0.9–1.0 eV. At the same time the luminescence quenching was irreversible. Its intensity did not recover when the samples were kept in the dark at room temperature. In this case, there are two processes leading to degradation of Ag${}_{2}$S/TGA QD luminescence intensity. The first is photolysis of Ag${}_{2}$Snanocrystals, described in detail in [18].

The second is process associated with a structural change in the QD interface under the action of exciting radiation since QDs luminescence properties are very sensitive to the interface structure, which is determined by synthesis methods. The restructuring/destruction reactions of surface ligands usually modify the physicochemical states of QDs surface atoms and dramatically change various QDs properties, including the photostability of QD surface atoms, resistance to the oxidative dissolution process, and tendency to coagulation/deposition. The effect of exciting radiation on thiol-coated QDs can lead to its photodestruction. In particular, the photodissociation of the C–OH-bond for TGA with the formation of α-thiol-substituted acyl radical (S-CH${}_{2}$-CO·) is known [50]. A similar photochemical reaction is supported by our FTIR spectra of Ag${}_{2}$S/TGA QD samples subjected to photo-exposure (Figure 2d). FTIR spectrum of QDs with exciton luminescence is significant changed. A change in the high-frequency form of the OH-banding mode is observed in the region of 3600–3500 cm${}^{-1}$. After exposure, a peak arises from the edge from this band near 3600 cm${}^{-1}$ due to vibrations of free or weakly bound OH-groups. A change in the intensities ratio of asymmetric (1574–1579 cm${}^{-1}$) and symmetric (1383–1388 cm${}^{-1}$) stretching vibrations of COO${}^{-}$-groups was observed. Probably it is caused by a change in the nature of the interaction with Ag${}_{2}$S QDs surface. A significant decrease in intensity is also observed in the band of stretching CO vibrations at 1222 cm${}^{-1}$, which indicates the destruction of this bond. Exposure to exciting radiation in this case leads to photodestruction of the thiol coating.

## 4. Conclusions

In this work the new data were obtained. It provides the situation understanding, related to the ambiguity of interpretation of TGA FTIR spectra on the whole. These data show that TGA molecule FTIR spectrum has a complex structure, strongly dependent on the concentration and pH value of solution. Based on experimental and calculation data, it is shown that the main manifestations of TGA molecules dimerization in FTIR spectra due to the formation of hydrogen bonds between them are bands behavior, belonging to the OH stretch as in COOH fragment, participating in the TGA dimer formation (3320 cm${}^{-1}$ and 2980 cm${}^{-1}$) and water molecules, located near TGA molecules and providing the formation of H-bond bridges, but which are less bounded with TGA molecules (3440 cm${}^{-1}$). In the region of these groups bending vibrations, the dimerization is confirmed by appearance of peak at 1714 cm${}^{-1}$ and feature at 1700 cm${}^{-1}$, characterizing the cyclic dimer and vibrations with peak at 900 cm${}^{-1}$. The band with peak at 1400 cm${}^{-1}$, associated with COH bending vibration and overlapping with the CH${}_{2}$ bending vibration, as well as complex vibration, including the CH${}_{2}$ wagging and twisting vibrations and C-O stretch at 1187 cm${}^{-1}$ and 1294 cm${}^{-1}$ are characteristic only for dimers and their specific symmetry. When pH value increases, the intensity of these bands decreases significantly up to their full leveling, which indicates dTGA and ddTGA. In addition, bands, corresponding to the stretching vibrations of carboxylate anion (1587 and 1414, 1412 cm${}^{-1}$) appear. The SH stretch at 2558 cm${}^{-1}$ disappears. The intensities of the bending vibrations decrease at 1025, 759 cm${}^{-1}$. And band at 629 cm${}^{-1}$ is completely leveled with the formation of carboxylate dianion. A mutual analysis of the experimental data and theoretical calculations results showed that the band in the region of 1640 cm${}^{-1}$, which is manifested as a high-frequency feature of $\nu_{as}$COO${}^{-}$ carboxylate anion peak (1565–1587 cm${}^{-1}$) under increasing in pH value belongs to δ(OH) in H${}_{2}$O.

Based on the obtained data for TGA various forms an unambiguous interpretation of TGA FTIR spectra under the conditions of passivation of silver sulfide quantum dots with fundamentally different luminescent properties was performed. The main mechanisms of molecular adsorption on QDs surface were determined. For samples synthesized without the use of Na${}_{2}$S, with exciton luminescence in the region of 620 nm and half-width less than 50 nm, adsorption of TGA molecules by two functional groups is characteristic. For colloidal QDs Ag${}_{2}$S/TGA solutions obtained using Na${}_{2}$S as a sulfur source with luminescence in the region of 880 nm of recombination nature, the adsorption of TGA molecules is carried out mainly by the thiol group. It was found that IR luminescence photodegradation is also accompanied by changes in the thioglycolic acid molecules, which capped Ag${}_{2}$S QDs. In the case of Ag${}_{2}$S QDs with exciton luminescence, the degradation process is non-reversible. It is accompanied by TGA photodegradation with the formation of α-thiol-substituted acyl radical (S-CH${}_{2}$-CO${}^{•}$) TGA.

## Figures and Tables

**Table 1:** Experimental and calculated data on characteristic modes of TGA FTIR bands in variously configurations.

TGA (98%)	TGA (pH = 8)	TGA (pH = 10)	TGA (Figure 1a)	TGA (Figure 1b)	TGA (Figure 1c)	TGA (Figure 1d)	TGA (Figure 1e)	Ag${}_{2}$S ($\lambda_{l}\mathrm{um}$ = 620 nm)	Ag${}_{2}$S ($\lambda_{l}\mathrm{um}$ = 880 nm)	Interpretation
3445	3321	3325		3486				3314	3370	ν(OH) in H${}_{2}$O near TGA
3220 2980	non-res 2963	non-res	3505	3256 3013 2976				3263	3228	ν(OH) in TGA COOH
2935 2882(sh)	2928 2900	non-res	3033 2948	2954 2900	2964 2914	2976 2933	2972 2929	2960 2923 2852	2987 2972 2914	$\nu (CH_{2})$
2567	2558	2558	2576	2559	2479		2534			ν(SH)
2662								2766 2620		overtone and combination bands of ν(C−O) at 1294 cm${}^{-1}$ and δ(COH) at 1400 cm${}^{-1}$
1714			1793	1722				1788 1727	1743	$\nu_{as}\left(C=O\right)+\delta \left(COH\right)$
1700(sh)				1685						$\nu_{s}\left(C=O\right)+\delta \left(COH\right)$
1640(sh)	1645(sh)	1646(sh)		1621				1679	1678	δ(OH) in H${}_{2}$O near TGA
	1587	1567			1594	1570	1580	1581	1574	$\nu_{as}\left(COO^{-}\right)+\delta \left(CH_{2}\right)$
1400			1346	1492 1478 1460 1448		1436				$\delta \left(CH_{2}\right)+\delta \left(COH\right)+\nu \left(C-O\right)$
	1414	1388			1339	1332	1376	1384 1359	1386	$\nu_{s}\left(COO^{-}\right)$
1294				1303				1222	1219	$\nu \left(C-O\right)+\omega \left(CH_{2}\right)$
	1237	1230	1257	1271 1268	1225	1204	1226			$\omega (CH_{2})$
1187	1213			1159 1151				1163		$\nu \left(C-O\right)+tw\left(CH_{2}\right)$
		1130	1121		1132	1131	1139	1126	1076	$tw(CH_{2})$
993	non-res	1025	952	1011 992	911		951	924		δ(SH)
900	908	923	894	974 929	919	883	899	909	898	γ(OH) in COOH or $\rho (CH_{2})$
758	759	759	752	838 808 783 746				787	770	$\rho \left(CH_{2}\right)+\delta \left(SH\right)$
668	672	672		675 662		673		700	695	δ(OCO)+ν(C−S)
	629	629		616	601		648	678		δ(OCO)+δ(SH)
577		595	672 587 521	580 575	572	537	577	570		$\delta (CH_{2})$ or δ(OCO)

* Non-res - the band is not resolution according the Rayleigh criterion.

## Abbreviations

The following abbreviations are used in this manuscript:

MDPI	Multidisciplinary Digital Publishing Institute
DOAJ	Directory of open access journals
TGA	Thioglycolic acid
QDs	Quantum dots
FTIR spectra	Fourier-transform infrared spectra
